# Severe white matter damage in *SHANK3* deficiency: a human and translational study

**DOI:** 10.1002/acn3.50959

**Published:** 2019-12-02

**Authors:** Sarah Jesse, Hans‐Peter Müller, Michael Schoen, Harun Asoglu, Juergen Bockmann, Hans‐Juergen Huppertz, Volker Rasche, Albert C. Ludolph, Tobias M. Boeckers, Jan Kassubek

**Affiliations:** ^1^ Department of Neurology Ulm University Ulm Germany; ^2^ Institute for Anatomy and Cell Biology Ulm University Ulm Germany; ^3^ Swiss Epilepsy Clinic Hospital Lengg Zurich Switzerland; ^4^ Core Facility Small Animal MRI Ulm University Ulm Germany; ^5^ DZNE Site Ulm Germany

## Abstract

**Objective:**

Heterozygous SHANK3 mutations or partial deletions of the long arm of chromosome 22, also known as Phelan–McDermid syndrome, result in a syndromic form of the autism spectrum as well as in global developmental delay, intellectual disability, and several neuropsychiatric comorbidities. The exact pathophysiological mechanisms underlying the disease are still far from being deciphered but studies of *SHANK3* models have contributed to the understanding of how the loss of the synaptic protein *SHANK3* affects neuronal function.

**Methods and results:**

Diffusion tensor imaging‐based and automatic volumetric brain mapping were performed in 12 SHANK3‐deficient participants (mean age 19 ± 15 years) versus 14 age‐ and gender‐matched controls (mean age 29 ± 5 years). Using whole brain–based spatial statistics, we observed a highly significant pattern of white matter alterations in participants with *SHANK3* mutations with focus on the long association fiber tracts, particularly the uncinate tract and the inferior fronto‐occipital fasciculus. In contrast, only subtle gray matter volumetric abnormalities were detectable. In a back‐translational approach, we observed similar white matter alterations in heterozygous isoform–specific Shank3 knockout (KO) mice. Here, in the baseline data sets, the comparison of Shank3 heterozygous KO vs wildtype showed significant fractional anisotropy reduction of the long fiber tract systems in the KO model. The multiparametric Magnetic Resonance Imaging (MRI) analysis by DTI and volumetry demonstrated a pathology pattern with severe white matter alterations and only subtle gray matter changes in the animal model.

**Interpretation:**

In summary, these translational data provide strong evidence that the *SHANK3*‐deficiency–associated pathomechanism presents predominantly with a white matter disease. Further studies should concentrate on the role of *SHANK3* during early axonal pathfinding/wiring and in myelin formation.

## Introduction

Phelan–McDermid Syndrome (PMS) is an orphan neurodevelopmental disorder with presumably several thousand participants worldwide. Due to numerous but mostly unspecific symptoms which include autistic behavior in up to 80%,^1^, ^2^, ^3^ global developmental delay, intellectual disability, severe speech impairment, reduced motor function, the syndrome is regarded as highly underdiagnosed, supported by the fact of an approximately 2% prevalence in patients with genetic autism spectrum disorders (ASD) with cognitive deficits.^1^, ^4^

The diagnosis is based on genetic investigations that feature the disease to be predominantly of de novo origin.^5^ Here, the genotype of most participants is characterized by loss or mutation of one copy of *SHANK3* localized on the distal part of chromosome 22q13 that – together with several other genes^6^ – codes for a scaffolding protein of the postsynaptic density of excitatory synapses, associated with reduced synaptic transmission and plasticity.^7^ *SHANK3* presumably is in the center of the underlying pathophysiology as isolated *SHANK3* loss or mutations lead to a phenotype very similar to 22q13.3 deletion syndrome.^3^, ^8^ On this pathophysiological basis, the syndrome is considered to be a disease of synapses and is considered to be part of the synaptopathy‐family.^9^, ^10^, ^11^

So far, single cases or case series of standard MRI in patients with *SHANK3* deficiency reported a variety of morphological intracerebral abnormalities.^3^, ^12^, ^13^, ^14^, ^15^ Most of these previous case studies and case series only used unsystematic approaches by visual MRI inspection resulting in a variety of gray and white matter structures found to be abnormal. However, based on the hypothesis that white matter alterations might constitute the link between symptoms of autism spectrum, cognitive deficits, motor impairment, and speech, we focused the current neuroimaging study on the white matter microstructure in SHANK3 deficient participants by diffusion tensor imaging (DTI). In addition, we performed a fully automatic volumetric analysis in order to quantify the hypothesized regional white matter changes in comparison with any changes of gray matter structures in a systematic whole brain‐based approach.

In a recent study, the first described primate *SHANK3*‐deficient model was analyzed with MRI and, by intrinsic functional connectivity MRI analysis, long fiber tract hypo‐ and short fiber tract hypo‐ and hyperconnectivity was observed, as correlates of an impaired default mode network.^16^

It is still on debate how the loss of one copy of SHANK3 leads to a variety of neurological and psychiatric symptoms. Since white matter alterations might constitute the link between symptoms of autism spectrum,^17^ cognitive deficits,^18^ motor impairment,^19^ and speech^20^ and given that structural connectivity analysis might be one way to unravel the in vivo morphological phenotype in autism spectrum disorders,^21^ we investigated the white matter microstructure in SHANK3‐deficient participants by diffusion tensor imaging (DTI). Then, based on the genetic background of the syndrome – we addressed the question whether microstructural pathologies in white matter areas could be traced back to SHANK3, as there are several genes lost in 22q13 deletions^22^ and cases are reported with a similar clinical picture and deletions on chromosome 22q13 without SHANK3 haploinsufficiency.^23^ To that end, participants were included with mutations within the SHANK3 gene only. Finally, to investigate the presence and extent of white matter changes in a back‐translational approach, we applied DTI analyses to a Shank3 (heterozygous ProSAP2/Shank3*αβ*+/−) knockout mouse model for human SHANK3 deficiency participants. Prosap2/Shank3 mutant mice were generated on a C57BL/6 strain background. The targeting strategy of the isoform‐specific knockout (KO) has been described from our laboratory by Schmeisser et al.^24^ This model shows typical autism‐like behavior such as excessive grooming and was analyzed before together with a non‐genetic autism mouse model for structural alterations of brain regions. This animal model is well‐established and displays heterozygosity for Shank3 as do the participants: several publications have shown that loss of SHANK3 in animal models is associated with core features of Phelan McDermid (PMD) syndrome.^24^, ^25^

## Material and Methods

### Participants

#### Participants and controls

All 12 participants (mean age 19 ± 15 years, range 2–57 years, six males/six females) had a genetically proven diagnosis of PMS. Genetic diagnosis revealed terminal deletions of chromosome 22q13 including *SHANK3*, one intragenic base pair duplication leading to a stop codon in *SHANK3* with loss‐of‐function, one balanced translocation with breakpoint in *SHANK3* and loss‐of‐function as well as one heterozygous point mutation in *SHANK3* (Table 1).

**Table 1:** Demographic and genetic data as well as clinical characterization of the patient cohort.

Patient no.	Age (years)		Gender	Genetic results
1	16		Female	Terminal deletion 22q13.31 (6.09 Mb)
2	3		Female	Terminal deletion 22q13.31 (3.1 Mb)
3	23		Female	Intragenic 1 base pair duplication, leading to a frameshift and stop codon in *SHANK3*, loss‐of‐function
4	17		Male	Terminal deletion 22q13.33 (111 Kb)
5	26		Female	Terminal deletion 22q13.33
6	17		Male	Heterozygous *SHANK3* point mutation
7	22		Male	Terminal deletion 22q13.33 (69 Kb)
8	58		Male	Balanced translocation under involvement of chromosome 1 and 22, breakpoint in *SHANK3*, loss‐of‐function
9	18		Male	Terminal deletion 22q13.33 (3 Mb)
10	2		Male	Terminal deletion 22q13.33 (3.3 Mb)
11	2		Female	Terminal deletion 22q13.33 (5.7 Mb)
12	19		Female	Terminal deletion 22q13.3

Patient no.	Motor function	Speech	Cognition	Autism	Autism spectrum	Dysmorphic signs
1	Moderate	Moderate	Moderate	n.a.	yes	None
2	Mild	Moderate	Moderate	no	no	None
3	Mild	Severe	Severe	n.a.	n.a.	Mild
4	Moderate	Moderate	Moderate	yes	n.a.	Moderate
5	Moderate	Mild	Mild	n.a.	yes	None
6	Moderate	Moderate	Moderate	n.a.	yes	Mild
7	Mild	Mild	Moderate	no	no	None
8	Mild	Mild	Moderate	n.a.	yes	None
9	Moderate	Severe	Moderate	yes	n.a.	Moderate
10	Moderate	Severe	Severe	n.a.	n.a.	Moderate
11	Moderate	Severe	Moderate	n.a.	yes	Mild
12	Mild	Moderate	Moderate	n.a.	yes	Mild

Mb, megabases; n.a., not available; Kb, kilobases; SHANK, SH3 and multiple ankyrin repeat domains 3.

Developmental stages and clinical characteristics of participants were classified for motor skills, speech, cognition, autism and autism spectrum symptoms, and morphological signs in “mild, moderate and severe” (Table 1). As nearly all of the patients with Phelan–McDermid syndrome reveal cognitive and speech impairment as well as symptoms of the autism spectrum, a lot of intelligence tests lack reliability and validity for children/adults with these symptoms. For this reason, cognition was classified according to the approach of the Diagnostic and Statistical Manual of Mental Disorders (DSM‐5)^26^ by a child‐ and adolescent psychiatrist. Here the classification of severity is not based on IQ testing and IQ points but on assessment of the adaptive functioning in conceptual, social, and practical domains which represents a feasible procedure for older patients with a stage of development of more than 3 years.

Autism and autism spectrum was diagnosed by using the Autism Diagnostic Interview (ADI) and the Autism Diagnostic Observation Schedule (ADOS)^27^ when applicable, whereas the youngest patients were classified only by clinical impression,^28^, ^29^ as these tests are not validated for the youngest even when they reveal a comorbid speech impairment.

In the context of this syndrome, it is obligatory for each patient to achieve MRI once. For the enrollment process, we included all participants with a genetically proven Phelan–McDermid syndrome who were admitted to our dedicated outpatient clinic, who had no MRI so far, and whose relatives/parents gave their consent to perform analgosedation. There were no specific exclusion criteria except from general factors of incompatibility with the procedures of the MRI scanning.

Participants were compared to 14 and gender‐matched controls in a similar age range, with no history of neurological or psychiatric disorders or any other medical condition (mean age 29 ± 5 years, range 19–37 years, six males/eight females). The group comparison concerning age and gender by Kruskal–Wallis test resulted in a *P*‐value of 0.05 and 0.59, respectively, indicating no major differences for the subject groups. All subjects or their caregivers gave written informed consent for the study protocol according to institutional guidelines, which had been approved by the Ethics Committee of Ulm University, Germany (reference# 321/16) and which were consistent with the declaration of Helsinki.

#### Animals

All animal experiments were performed in compliance with the guidelines for the welfare of experimental animals issued by the Federal Government of Germany, the National Institutes of Health and the Max Planck Society. The experiments in this study were approved by the review board of the country Baden‐Württemberg (regional board Tübingen) and the local ethics committee at Ulm University (reference #1239). All animals were bred and mated in the animal facility of Ulm University.

*Prosap2/Shank3* mutant mice were generated on a C57BL/6 strain background. The targeting strategy of the isoform‐specific knockout has been described from our laboratory by Schmeisser et al.^24^ In synopsis, exon 11 in the SH3 domain was deleted, thereby resulting in a translational stop sequence. The western blot phenotype with reduced *α*‐ and *β*‐isoforms referred to the genotype *ProSAP2/Shank3αβ^+/^* ^−^, which has been named as *Shank3*‐isoform–specific heterozygous KO hereafter.

### MRI scanning

#### MRI acquisition in humans

MRI scanning was performed on a 1.5 Tesla Magnetom Symphony (Siemens Medical, Erlangen, Germany); the DTI study protocol consisted of 52 volumes (64 slices, 128 × 128 pixels, slice thickness 2.8 mm, pixel size 2.0 × 2.0 mm), representing 48 gradient directions (*b* = 1000 s/mm^2^) and four scans with *b* = 0, time of echo (TE) and time of repetition (TR) were 95 and 8000 msec. Further scanning included a T2‐weighted data set (Fluid Attenuated Inversion Recovery/FLAIR) with 40 coronal slices, TR/TE 6180/112 msec) of 3.0 mm thickness, 0.45 × 0.45 mm in‐plane resolution and 512 × 448 voxels matrix dimension and a T1‐weighted imaging (MPRAGE) consisting of 144 sagittal slices of 1.2 mm thickness, 1.0 × 1.0 mm in‐plane resolution and 256 × 248 voxels matrix dimension. For MRI scanning, all participants received short analgesic sedation under monitoring of the vital parameters by an anesthetist, using propofol in combination with esketamine or midazolam.

#### MRI acquisition in animals

Animal imaging was performed with a 11.7T small bore animal scanner (Biospec 117/16, Bruker, Ettlingen, Germany). A cryogenic 1H two‐element transmit/receive mouse head coil (Cryo‐Probe, Bruker BioSpin, Ettlingen, Germany) was used for data acquisition. Imaging parameters of the optimized rapid diffusion prepared respiratory gated spin echo EPI imaging protocol were as follows: TE/TR 36.0/5000 msec, matrix 180 × 40, in‐plane resolution 102 × 102 *µ*m, 70 slices with a slice thickness of 250 *µ*m. Sixty‐four diffusion directions with *b* = 1000 s/mm^2^ and one unweighted *b* = 0 volume (standard gradient scheme as provided by the Bruker software), 1 signal average, were acquired, resulting in a total acquisition time of 22 min.

Data acquisition was performed under isoflurane anesthesia (5% for induction and 1.5% for maintenance). The animals were placed in a stereotactic head support (Bruker Biospin, Ettlingen, Germany) to immobilize the head. Body temperature was controlled by an integrated water‐based heating device. The body temperature of the mouse was monitored by a rectal temperature probe and respiration was monitored by a respiratory pillow positioned under the abdomen of the mouse. The breathing frequency was maintained at 75–80 cycles per min. The mice rapidly recovered (<5 min) after termination of anesthesia at the end of the MRI procedure.

In total, 12 *Shank3*‐isoform–specific heterozygous KO mice and 17 wildtype (wt) mice were scanned at baseline (4 weeks) with one follow‐up scan (9 weeks). DTI data of five additional *Shank3* isoform–specific heterozygous KO mice at baseline and seven *Shank3* isoform–specific heterozygous KO mice at follow‐up and seven wt mice at baseline, four wt mice at follow‐up did not pass quality control due to motion artifacts. In summary, seven *Shank3* isoform–specific heterozygous KO mice and 10 wt mice at baseline, as well as five *Shank3*‐ isoform–specific heterozygous KO mice and 13 wt mice at follow‐up were used for analysis. Volumetric data on these mice as well as on a prenatal zinc–deficient mouse model have recently been published by our group.^25^

### Data analyses

#### DTI analysis in humans

The postprocessing and statistical analyses were performed by use of the software platform *Tensor Imaging and Fiber Tracking* (TIFT).^30^ In order to spatially normalize the data to the Montreal Neurological Institute (MNI) stereotaxic standard space, study‐specific templates were created and MNI normalization was performed iteratively.^31^ From the normalized DTI data sets, fractional anisotropy (FA) maps were calculated for quantitative mapping of structural connectivity.^32^ In a consecutive step, an 8 mm (FWHM) Gaussian filter was applied for smoothing of FA maps within the whole brain–based statistical analysis (WBSS) in order to achieve a good balance between sensitivity and specificity. For smoothing, the fact that filter size influences results of DTI data analyses^33^ requires application of the matched filter theorem which states that the width of the filter used to process the data should be tailored to the size of the difference one expects.^34^ Unbiased WBSS was performed by comparing voxelwise FA values of PMS patients and controls at the group level (Student's *t*‐test). FA maps of PMS patients and controls were not age‐corrected as no correction parameters were available for the 2‐year‐old PMS patients.

Tractwise fractional anisotropy statistics (TFAS) was performed by statistically comparing the FA values between the two subject groups in a given tract system (Student's *t*‐test).^35^ We focussed on the uncinate fasciculus and the inferior fronto‐occipital fasciculus together with the corticostriatal pathway, based on the data‐driven approach that these tracts were identified by WBSS to be significantly altered in FA. Consequently, a tract‐of‐interest (TOI) analysis allowed for quantification of microstructural alterations in these tract systems.^36^

#### Atlas‐based volumetry in humans

The fully automated Atlas‐based volumetry (ABV) method is based on algorithms of SPM (Wellcome Department of Imaging Neuroscience, London, United Kingdom; http://www.fil.ion.ucl.ac.uk/spm) and masks derived from a probabilistic brain atlas provided by the Laboratory of Neuroimaging (LONI) at the University of California, Los Angeles, USA (LONI Probabilistic Brain Atlas (LPBA40); http://www.loni.ucla.edu/Atlases) and has been fully described previously.^35^ For the purpose of the current study, gray and white matter volumes were measured. Volumetric measures were corrected for intracranial volumes.^37^, ^38^

#### DTI analysis in animals

Data processing was also performed with the *Tensor Imaging and Fiber Tracking* (TIFT) software package,^30^ which has been successfully applied to animal DTI group studies.^39^, ^40^ Recorded data were transformed into a 50 *µ*m iso‐grid (nearest neighbor interpolation) in order to minimize partial volume effects. The slice thickness to in‐plane resolution ratio of 1.6 as well as the recorded brain grid were in analogy in human DTI studies since the transformation to an iso‐grid of 50 *µ*m corresponds to an iso‐grid of 1 mm in human studies. Spatial normalization to a stereotaxic standard space was performed using a study‐specific b0‐template and an FA‐template.^40^ Motion artifacts were eliminated in each data set separately by a dedicated quality check procedure. During the iterative normalization process, scanner‐ and sequence specific b0‐ and FA‐templates were created by arithmetically averaging data sets of all mice after linear transformation according to manually set landmarks identified using a stereotaxic mouse atlas.^41^ After this first iteration, data were nonlinearly normalized during a second iteration step in order to further optimize the normalization matrices. This process was iteratively repeated until the correlation between the individual FA maps and the FA template was >0.7, which was achieved after two iterations. Motion artifacts were eliminated in each data set separately by a dedicated quality check procedure.^42^ FA maps of each data set were calculated and were smoothed with a Gaussian filter of 200 *µ*m full‐width‐at‐half‐maximum. The filter size, which is about 2–3 times the recording voxel size, provides a good balance between sensitivity and specificity. WBSS and TFAS were applied in analogy to human DTI data.^39^

## Results

### Whole brain–based spatial statistics of FA maps in humans

**Figure 1:**
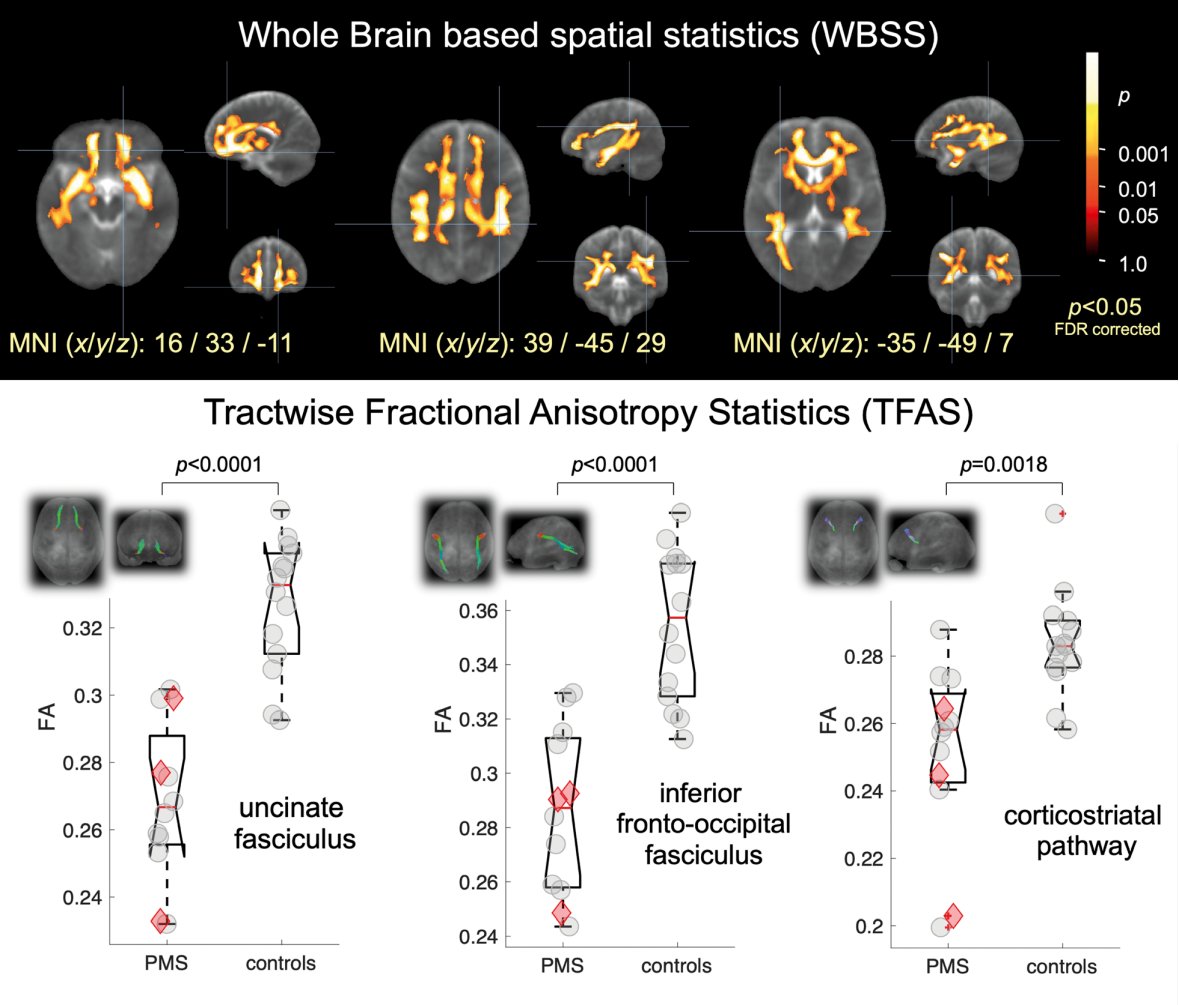
**Upper panel:** Whole brain–based spatial statistics (WBSS) of FA maps at the group level for 12 PMS patients versus 14 controls. WBSS of FA maps demonstrates multiple clusters of regional FA reductions at *P* < 0.05 (corrected for multiple comparisons, false‐discovery‐rate (FDR) corrected). **Lower panel:** Tractwise fractional anisotropy statistics (TFAS) results for comparison of PMS patients to controls in the respective tract systems – projectional views of tract reconstructions as inlays. The red icons indicate participants with *SHANK3* only mutations. FA, fractional anisotropy; FDR, false‐discovery‐rate; MNI, Montreal Neurological Institute; PMS, Phelan–McDermid syndrome; WBSS, whole brain–based spatial statistics.

The unbiased whole brain‐based comparison at the group level for *SHANK3*‐deficient participants versus controls demonstrated multiple clusters of regional FA decreases at *P* < 0.000001 (corrected for multiple comparisons, FDR). The pattern of the microstructural alterations consisted of two large interconnected clusters: first, 194,462 voxels, maximum at MNI (*x/y/z)* 12/23/−8 and second, 4145 voxels, MNI 2/−16/−34. The widely observed FA reduction pattern spread from the frontal and limbic lobes to the temporal and parietal lobes (Fig. 1 upper and lower panel).

### Differences of FA in the tract systems in humans

Based on the pattern of microstructure alterations as demonstrated by WBSS, a TFAS/TOI‐based analysis of the FA differences was performed in order to specify the involved tract systems. Specific regional FA alterations were located in the uncinate tract (*P* = 0.000003), in the inferior fronto‐occipital fasciculus (*P* = 0.00002), and in the corticostriatal pathway (*P* = 0.002) (Fig. 1 upper and lower panel).

Since there were different age ranges in the patient and control group, statistical analyses were repeated without the three youngest participants (age of 2 or 3 years) to control for an age‐related bias; this subgroup analysis confirmed the WBSS results (Fig. S1) as well as the TFAS results (Table S1).

### Atlas‐based volumetry results in humans

**Figure 2:**
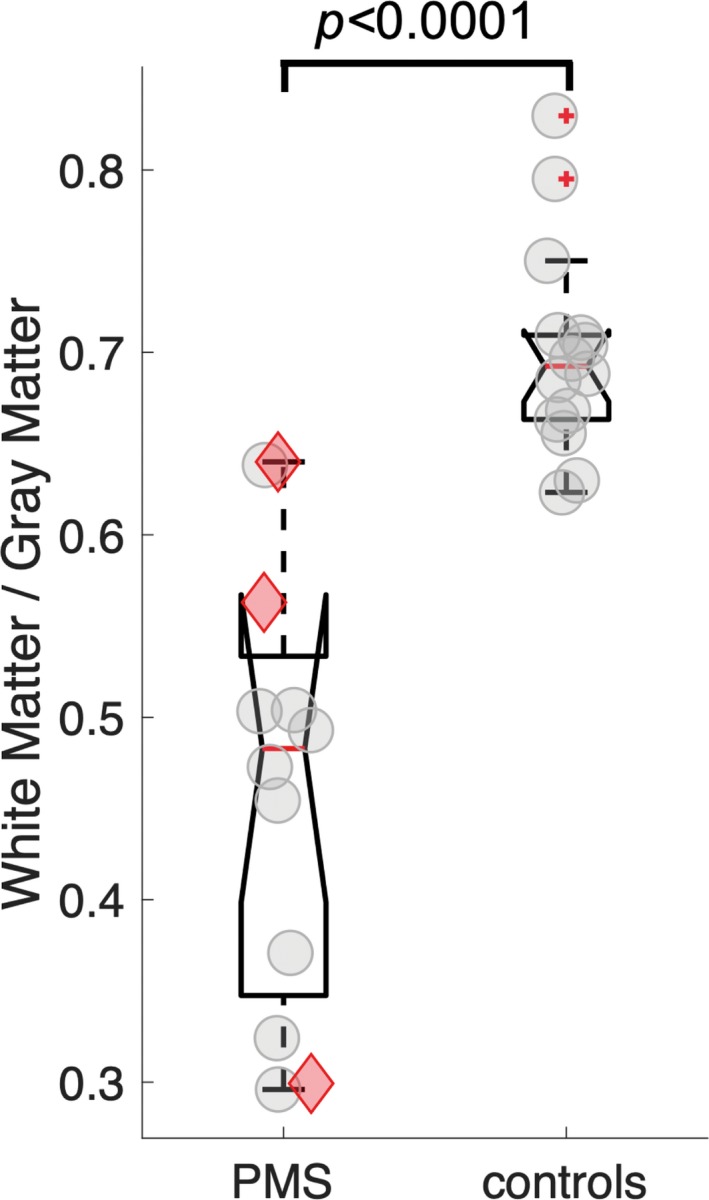
White matter to gray matter ratio of 12 PMS patients and 14 controls. Results were obtained from atlas‐based volumetry (ABV). PMS 0.49 ± 0.11, controls 0.70 ± 0.06, *t*‐test *P* = 0.00001. Marked in red are the *SHANK3* only PMS patients. ABV, atlas‐based volumetry; PMS, Phelan–McDermid syndrome; *SHANK3*, SH3 and multiple ankyrin repeat domains 3.

The investigation of intra cranial volume ICV‐corrected gray matter (GM) and white matter (WM) volumes of *SHANK3*‐deficient participants compared to controls showed significantly reduced WM substructure volumes, whereas no significant differences were found for GM substructure volumes (Table 2). The volumes of tract systems (bold in Table 2) also showed a significant reduction for *SHANK3*‐deficient participants. Consequently, the WM to GM ratio was highly significantly reduced (*P* = 0.0001) in the patient group (Fig. 2) – a result that cannot be explained by an age‐related effect.

**Table 2:** Group–averaged white matter (WM) and gray matter (GM) structures of 12 PMS participants versus 14 controls obtained from atlas‐based volumetry (ABV).

	PMS (mean)	PMS (SD)	Controls (mean)	Controls (SD)	*P*‐values (*t*‐test)	
Globals						
Brain	1129.4	128.8	1198.8	87.6	0.13	
Gray matter (GM)	774.1	85.9	705.0	46.5	0.024	
White matter (WM)	355.2	85.1	493.8	51.6	0.00012	
Cerebrospinal fluid	249.5	64.3	271.1	40.2	0.33	
Intracranial volume (ICV)	1378.9	151.0	1469.9	107.0	0.096	
WM/GM	0.46	0.12	0.70	0.06	0.000014	
Substructures (ICV‐corrected)						
Cerebellum WM	19.4	3.1	24.8	1.7	0.000048	**WM**
Cerebrum WM	313.2	54.0	417.5	19.7	0.000021	
Temporal lobe WM	44.1	7.1	58.9	3.2	0.000009	
Frontal lobe WM	102.9	19.3	140.4	9.8	0.000017	
Parietal lobe WM	55.6	11.2	77.0	3.5	0.000025	
Occipital lobe WM	40.5	8.0	52.9	2.7	0.000180	
**Superior longitudinal fascicle**	2.4	0.3	3.0	0.1	0.000042	
**Optic radiation**	16.8	2.1	20.6	0.7	0.000042	
**Superior occipitofrontal fascicle**	1.1	0.2	1.4	0.1	0.000061	
**Uncinate fascicle**	0.2	0.0	0.3	0.0	0.00015	
**Corticospinal tract**	16.6	2.9	21.1	1.1	0.00022	
**Inferior occipitofrontal fascicle**	0.6	0.1	0.7	0.1	0.00033	
**Acoustic radiation**	1.4	0.2	1.7	0.1	0.00067	
Putamen GM	9.8	0.9	8.3	0.5	0.000057	
Insula GM	20.1	2.0	17.2	0.7	0.000269	
Cerebellum GM	107.4	7.9	99.2	7.1	0.012	**GM**
Cerebrum GM	668.4	84.2	560.6	28.0	0.00096	
Cerebral cortex	646.1	83.8	540.6	28.0	0.0011	
Temporal lobe GM	158.8	18.3	135.7	6.5	0.0010	
Frontal lobe GM	221.7	26.0	188.9	10.1	0.0010	
Parietal lobe GM	130.6	22.2	105.5	9.0	0.0025	
Occipital lobe GM	91.2	14.6	74.2	4.2	0.0019	
Striatum	19.0	1.6	16.9	1.2	0.0010	
Medulla	4.4	0.5	4.7	0.3	0.035	
Brainstem	28.3	2.8	30.3	2.1	0.052	
Pons	14.1	1.8	15.3	1.4	0.071	
Midbrain	9.8	0.8	10.3	0.6	0.072	
Hippocampus and amygdala	11.4	1.1	10.8	0.5	0.082	

Substructure volumes (in mm^3^) are sorted for WM and GM. Defined fiber tracts are in bold. *P*‐values < 0.001 (reduced volume of the PMS group) are marked in yellow. GM, gray matter; ICV, intracranial volume; PMS, Phelan–McDermid syndrome; SD, standard deviation; WM, white matter.

### Whole brain–based spatial statistics in *Shank3* heterozygous KO mice

The experimental design in the mouse model allowed two sets of analyses [36]: firstly, the cross‐sectional comparison of *Shank3* isoform–specific heterozygous KO cohorts and wt at baseline and at follow‐up and, secondly, the longitudinal comparison of *Shank3*‐isoform–specific heterozygous KO mice at baseline and follow‐up.

**Figure 3:**
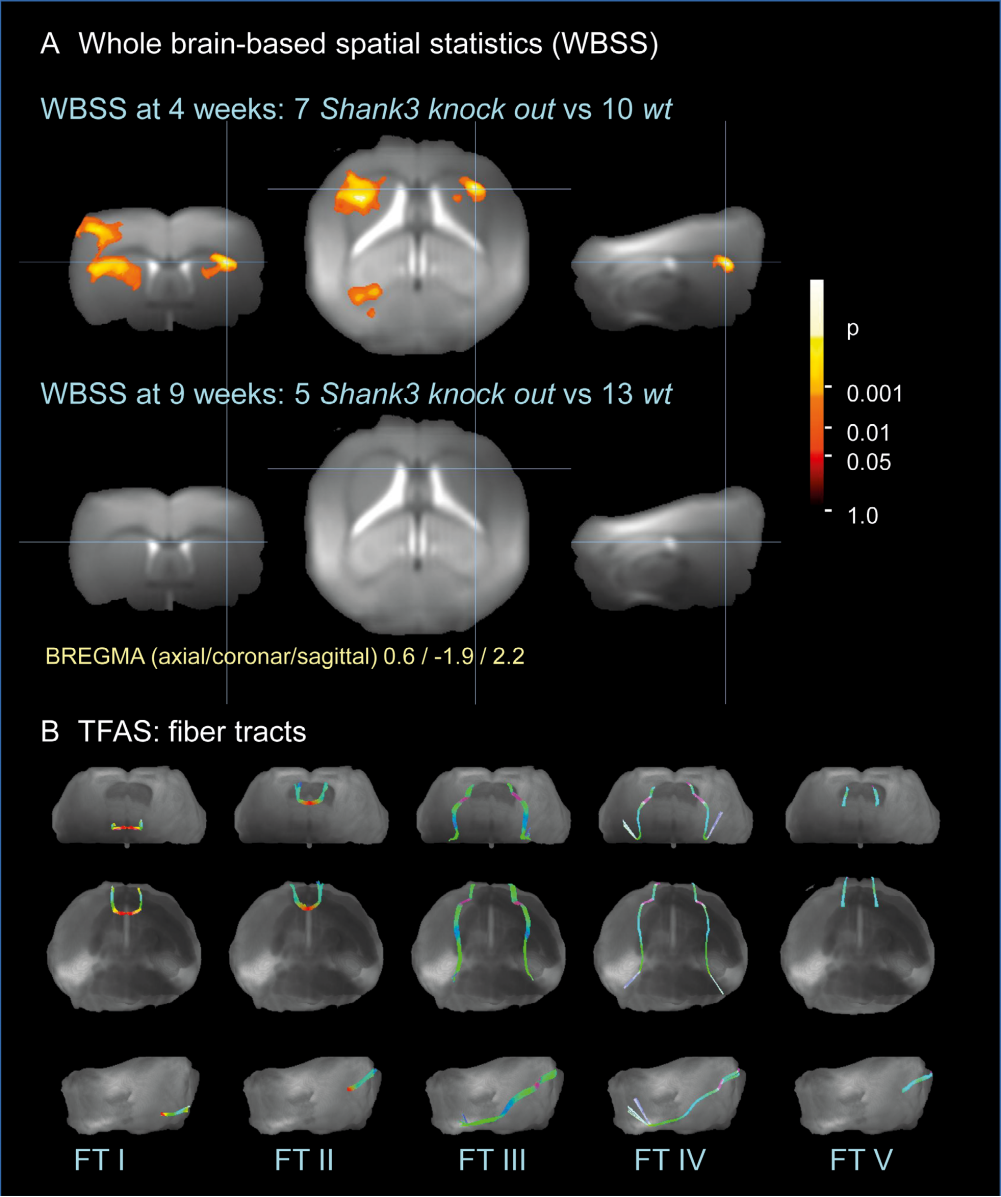
(A) WBSS of *Shank3* isoform–specific heterozygous KO versus wt at 4 weeks and at 9 weeks. (B) projectional views of fiber tracts, representative for short (FT I, II and V) and long connections (FT III and IV). FT I: part of CC connecting frontal association cortices; FT II: anterior commissure, FT III: association fiber tract from motor cortex to entorhinal cortex; FT IV: fronto‐occipital association fiber tract connecting V1 and frontal association cortex; FT V: corticostriatal pathway. FT, fiber tract; KO, knockout; *Shank3*, SH3 and multiple ankyrin repeat domains 3; TFAS, tractwise fractional anisotropy; WBSS, whole brain–based spatial statistics; wt, wildtype.

At baseline (4 weeks of age), *Shank3* isoform–specific heterozygous KO compared to wt displayed significant regional FA reduction hemispherical in the agranular insular cortex and the caudoputamen and in the lateral periaqueductal gray matter. At follow‐up (9 weeks of age), no significant FA difference between *Shank3* isoform–specific heterozygous KO and wt could be observed. Consequently, an FA increase in the caudoputamen in *Shank3* isoform–specific heterozygous KO from 4 to 9 weeks was detected (Fig. 3A).

### Tractwise fractional anisotropy in specific tract systems in *Shank3* heterozygous KO mice

In *Shank3* heterozygous KO mice, the tractwise analysis of FA alterations in association fibers was performed in five tract systems, i.e., FT I, part of CC connecting frontal association cortices; FT II, anterior commissure, FT III, association fiber tract from motor cortex to entorhinal cortex; FT IV, fronto‐occipital association fiber tract connecting V1 visual cortex and frontal association cortex; FT V, corticostriatal pathway (Fig. 3B). In the baseline data sets, the comparison between *Shank3* isoform–specific heterozygous KO versus wt showed no differences in association fibers (FT I, FT II, FT V), whereas the long fiber tract systems (FT III, FT IV) revealed significant FA reduction from wt to *Shank3* isoform–specific heterozygous KO. In the follow‐up datasets, all fiber tract systems and also whole brain showed no significant FA alterations between *Shank3* isoform–specific heterozygous KO and wt (Table 3).

**Table 3:** *P*‐values as results of TFAS separating tracts with significant differences between *Shank3*‐isoform–specific heterozygous KO and wt at 4 weeks and at 9 weeks.

	FT I	FT II	FT III	FT IV	FT V	Whole brain
*wt* (4 weeks) versus *wt* (9 weeks)	0.63	0.06	0.14	0.53	0.71	0.94
*Shank3* versus *wt* (4 weeks)	0.52	0.21	0.002	0.002	0.70	0.004
*Shank3* versus *wt* (9 weeks)	0.84	0.94	0.66	0.82	0.23	0.99
*Shank3* (4 weeks) versus *Shank3* (9 weeks)	0.48	0.17	0.04	0.05	0.30	0.02

Significance is marked in yellow with *P* < 0.05. FT I: part of CC connection to frontal association cortices; FT II: anterior commissure; FT III: association fiber tract from motor cortex to entorhinal cortex; FT IV: fronto‐occipital association fiber tract connecting V1 and frontal association cortex; FT V: corticostriatal pathway. CC, corpus callosum; FT, fiber tract; *Shank3*, SH3 and multiple ankyrin repeat domains 3. TFAS, tractwise fractional anisotropy statistics; wt, wildtype.

## Discussion

### Main findings

The current human and back‐translational MRI investigations were based on the hypotheses that the clinical PMS phenotype might be influenced by microstructural pathologies in the brains of these participants that can be attributed to *SHANK3* deficiency and should therefore be reproduceable in our *Shank3* KO mouse model.

By postprocessing of DTI data and FA as a compound marker of white matter integrity, we obtained virtual dissections of white matter fiber bundles in vivo*.* The microstructural impairment showed a significant focus on long fiber tracts (i.e., uncinate fasciculus and inferior fronto‐occipital fasciculus) as well as the corticostriatal tract. In summary, the multiparametric MRI analysis by DTI and volumetry demonstrated a pathology pattern with severe white matter alterations which were clearly predominant compared with gray matter changes.

These results held for all individual participants, irrespective of gender, for both deletions and *SHANK3*‐specific mutations, clinical presentation, or age. Especially the prominent white matter involvement across all ages is remarkable, given the broad age heterogeneity of the study cohort, since previous DTI studies of participants with ASD using various technical approaches have suggested that ASD–related microstructural WM alterations differ apparently depending on the age of the individuals studied.^43^ Here, MRI data in ASD individuals featured less impairment of adults, whereas younger participants on the one hand and mouse models on the other hand showed more structural pathology.^44^, ^45^, ^46^ Therefore, we investigated our data with respect to the youngest and oldest of our participants and found adult PMS patients to show a tendency of less volume decrease in the white matter (data not shown) which is congruent with the mentioned literature and our *Shank3* KO mouse model showing similar white matter changes in young animals, whereas older animals did not present this damage.

As a consequence of these observations in our study and in ASD in general, one might assume compensatory effects that take place during maturation of the brain by reorganization of the gray and white matter compartments with a strengthening of distinct regional connectivity patterns and preserved structural integrity in key anatomical regions (like the prefrontal cortex, hippocampus, and corpus callosum).^47^

### Long fiber tract pathology in PMS patients

Both the uncinate fasciculus and the inferior fronto‐occipital fasciculus are long association connections which interconnect cortical regions in different lobes within the same hemisphere. The first one connects the inferior frontal gyrus and the orbital surface of the frontal lobe with anterior portions of the temporal lobe, the latter consists of fibers that connect the lateral parts of the frontal lobe with the inferior temporal and medial/lateral occipitotemporal gyri and with the occipital lobe.^48^ As such, they integrate multimodality functions of neocortical regions and are involved in language transformation. The uncinate tract as a connection between the temporal lobe to the prefrontal cortex plays a part in auditive speech‐procession, semantic long‐term memory as well as recall of contents out of the episodic long‐term memory.^49^ The inferior fronto‐occipital fasciculus acts in reading, attention, and visual processing by connecting the occipital cortex, temporo‐basal areas, and superior parietal lobe to the frontal lobe.^50^ The global white matter changes including these major association fiber tracts of the neocortices with their key integrative function in cognitive processing might represent a pathophysiological correlate for cognitive decline as well as expressive and receptive speech impairment in participants with *SHANK3* deficiency.

The delayed achievement of motor milestones seen in Phelan–McDermid Syndrome may be associated with white matter changes in the sensorimotor and associative fractions of the corticostriatal tract^51^ which also showed FA reductions, although less prominent than the long association fibers. Moreover, motor dysfunctions covered by the corticostriatal system (which is a multifunctional circuit addressing aspects of motor control, cognition, and motivation) may also be related to the disturbance in procedural memory.^52^ Investigation of the corticospinal tract system also revealed volume decrease and FA impairment (data not shown) but to a lesser extent than the corticostriatal system.

One could speculate that impaired migration of multipolar nerve cells in the developing brain may lead to hampered aggregation and differentiation of neuronal networks with the consequence of damaged axonal sprouting ending in impaired pathway and target selection of the axon, which does not find its neuronal counterpart leading to compromised synapse formation. In this context, the synaptopathy may be an additional or even secondary effect of a primarily axonal neurodevelopmental disorder.

### SHANK3 attribution

There is some uncertainty if *SHANK3* is the key gene responsible for the neurological findings as there are many genes lost in 22q13 microdeletions and there are a few cases presenting a “PMS phenotype,” combined with a 22q13 interstitial deletion but without *SHANK3* allele loss.^22^, ^23^ Otherwise, there is evidence that haploinsufficiency of *SHANK3* due to point mutations is sufficient to cause a broad range of features associated with PMS.^3^, ^53^ In our study, the same severe microstructural changes of white matter could be detected in those participants with *SHANK3* mutations as in the larger group featuring microdeletions of chromosome 22q13.3. This suggests that *SHANK3* haploinsufficency is sufficient to cause the white matter changes observed in our patient cohort.

### White matter alterations in SHANK3 model organisms

How can these results be integrated in the context of former data concerning white matter changes in *SHANK3‐*associated models? Apart from a few case reports showing routine MRI with white matter alterations in terms of impaired myelination but without investigation of the WM in detail, there are no human data on fiber tract integrity of PMS individuals. Indeed, there have been some studies in non‐*Shank3* autism mouse models^45^, ^46^ which demonstrated functional and structural alterations in circuits of the anterior and posterior forebrain.^54^, ^55^ A previous study reported largely disrupted functional connectivity and abnormal gray matter anatomy in prefrontal areas of homozygous *Shank3* KO mice, associated with socio‐communicative deficits. Interesting aspects to discuss with regard to our investigations is the fact that the mice in this study showed marked FA alterations in even older mice than our mice at 9 weeks of age. This might be traced back to the fact of more deleted isoforms in their model and that they analyzed homozygous KO.^56^ In addition to the mouse models, Zhou et al. described alterations of the default mode network by intrinsic functional connectivity in the MRI of *SHANK3*‐deficient macaques.^16^

### Limitations

There are some limitations of our study that should be addressed. The low sample size as well as the fact that we could investigate the participants only cross‐sectionally due to their clinical condition and ethical considerations, compromises the comparability to the animal model that was investigated in a longitudinal design. Moreover, our heterozygous model harbors one intact *Shank3* allele and the other allele is still capable of expressing remaining isoforms. This might explain why we observed white matter alterations only in the younger animals.

In the clinical study, the age range was enormous and only few participants were in their childhood. For ethical reasons, it is hardly possible to obtain comparably young healthy controls for an MRI measurement. On the other hand, it would be a pity to dispense the youngest representatives of such a rare disease. Against this background, the matching of age and sex in patient and control groups, as carried out in this study, and which, according to the Kruskal–Wallis test, showed no significant differences between the groups, appeared to be the best possible solution. In addition, the subgroup analysis carried out excluding the three youngest patients (2–3 years old) reproduced the overall results and, in our opinion, represents a valid approach for verification and confirmation.

It will be important to conduct longitudinal investigations of white matter alterations in PMS individuals in order to study progression or decrease of the pathology.

### Conclusion and outlook

PMS is presumed to be a synaptic disorder as the probable disease‐defining gene *SHANK3* codes for a synaptic protein of the postsynaptic density.^57^, ^58^ The results of the current study suggest an additional involvement of the SHANK3 protein in axonal path finding, fiber bundle integrity, and myelin formation given that we detected an impairment of the fiber integrity. To classify PMS also as a white matter disease is supported by our former studies that indicated SHANK family members not to be limited to the postsynaptic density but also to be expressed presynaptically in axonal as well as growth cone areas.^5^

In contrast to the highly significant white matter alterations, gray matter changes were only subtle for substructure volumes, and although the gray matter of the cerebrum was also observed to be reduced in PMS, the white matter volume reductions were much more pronounced as mirrored in the white matter/gray matter ratio.

For these reasons, it is time to take a closer look into the role of *SHANK3* not only at postsynaptic sites but also at presynaptic areas during early axonal pathfinding and in glial cells and to perform histological studies to understand the pathophysiological basis of the white matter damage, which could illustrate impaired connectivity of the long fiber tracts or altered myelination/demyelination processes with impaired axonal functionality. To get a more detailed impression of longitudinal follow‐up investigations of *SHANK3*‐deficient participants, DTI mapping might serve as a biological marker in larger multisite patient groups.

In summary, *SHANK3* function might be underestimated in its pivotal role in neuronal tissue. To our knowledge, this is the first report by advanced neuroimaging of massive white matter alterations in *SHANK3*‐deficient participants with PMS, which back‐translates with *Shank3*‐deficient model organisms. We suggest to further investigate the neurobiological role of *SHANK3* in axons and myelin formation and to correlate the MRI phenotype with the clinical phenotype to elucidate the role of this alteration in disease progression and, further, as a putative biological marker.

## Author Contributions

SJ contributed to the concept, design and evaluation of the study and acquired the human MRI data. HPM contributed to the evaluation of the human and animal MRI data. MS contributed to the concept, design, evaluation and critical reevaluation of the study. HA contributed to the evaluation of the study. JB contributed to the evaluation of the study. HJH contributed to the evaluation of the animal MRI data. VR contributed to the evaluation of the animal MRI data. ACL contributed to the evaluation of the study. TMB contributed to the concept, design and evaluation of the study and acquired the human MRI data. JK contributed to the concept, design and evaluation of the study and acquired the human MRI data.

## Conflict of Interest

The authors declare that there is no conflict of interest to disclose.

## Supporting information


**Table S1** **.** Upper panel: Cluster coordinates of FA reduction (WBSS) in nine adolescent and adult PMS patients (14–56 years old) versus nine matched controls. Lower panel: Results of TFAS of these nine PMS patients and nine controls. Significance is marked in yellow with *P* < 0.05.
**Figure S1** **.** Whole brain–based spatial statistics (WBSS) of FA maps at the group level for nine adolescent and adult PMS patients (14–56 years old) versus nine matched controls. WBSS of FA maps demonstrated multiple clusters of regional FA reductions at *P* < 0.05 (corrected for multiple comparisons, FDR).
