# Supplementary material for: Severe white matter damage in SHANK3 deficiency: a human and translational study
Source: Ann Clin Transl Neurol. 2019 Dec 2;7(1):46–58. doi: 10.1002/acn3.50959 (PMC6952316; doi:10.1002/acn3.50959)
Supplement: Supplementary file 1 — Table S1 . Upper panel: Cluster coordinates of FA reduction (WBSS) in nine adolescent and adult PMS patients (14–56 years old) versus nine matched controls. Lower panel: Results of TFAS of these nine PMS patients and nine controls. Significance is marked in yellow with P < 0.05. Figure S1 . Whole brain–based spatial statistics (WBSS) of FA maps at the group level for nine adolescent and adult PMS patients (14–56 years old) versus nine matched controls. WBSS of FA maps demonstrated multiple clusters of regional FA reductions at P < 0.05 (corrected for multiple comparisons, FDR). [file ACN3-7-46-s001.pdf]

Severe white matter damage in *SHANK3* deficiency: a human and translational study

Sarah Jesse[1*], Hans-Peter Müller[1], Michael Schoen[2], Harun Asoglu[2], Juergen Bockmann[2], Hans-Juergen Huppertz[3], Volker Rasche[4], Albert C. Ludolph[1,5], Tobias M. Boeckers[2,5], Jan Kassubek[1]

[1]Ulm University, Department of Neurology, Germany

[2]Ulm University, Institute for Anatomy and Cell Biology, Germany

[3]Swiss Epilepsy Clinic, Hospital Lengg, Zurich, Switzerland

[4]Core Facility Small Animal MRI, Ulm University, Ulm, Germany

[5]DZNE site, Ulm, Germany

***Corresponding author:**

PD Dr. Sarah Jesse

Department of Neurology, Ulm University

Oberer Eselsberg 45

D-89081 Ulm, Germany

Email: sarah.jesse@uni-ulm.de

Supplementary material

Supplementary Table 1: Upper panel: Cluster coordinates of FA reduction (WBSS) in 9 adolescent and adult PMS patients (14-56 years old) vs. 9 matched controls. **Lower panel:** Results of TFAS of 9 these PMS patients and 9 controls. Significance is marked in yellow with p <0.05

Abbreviations: FA = fractional anisotropy; MNI = Montreal Neurological Institute; PMS = Phelan-McDermid syndrome; TFAS = tractwise fractional anisotropy; WBSS = whole brain-based spatial statistics.

**WBSS**				
**cluster no.**	**R/L**	**MNI (x/y/z)**	**Size**	**p values**
1	L	-35 / 2 / -30	15254	< 0.000001
2	R	32 / -3 / 13	11463	< 0.000001
3	L	-41 / -43 / 0	3004	< 0.000001
4	R	42 / -38 / 29	1933	< 0.000001
5	R/L	0 / -27 / -33	1710	< 0.000001
6	L	-47 / -53 / 42	957	< 0.000001
7	R	6 / -8 / -32	661	< 0.000001
8	R	37 / -28 / 7	572	< 0.000001
**TFAS**				
		**PMS**	**Controls**	**p values**
uncinate fasciculus		0.283 ± 0.021	0.311 ± 0.021	0.0006
inferior fronto-occipital fasciculus		0.311 ± 0.021	0.351 ± 0.024	0.03
corticostriatal pathway		0.262 ± 0.015	0.279 ± 0.012	0.02

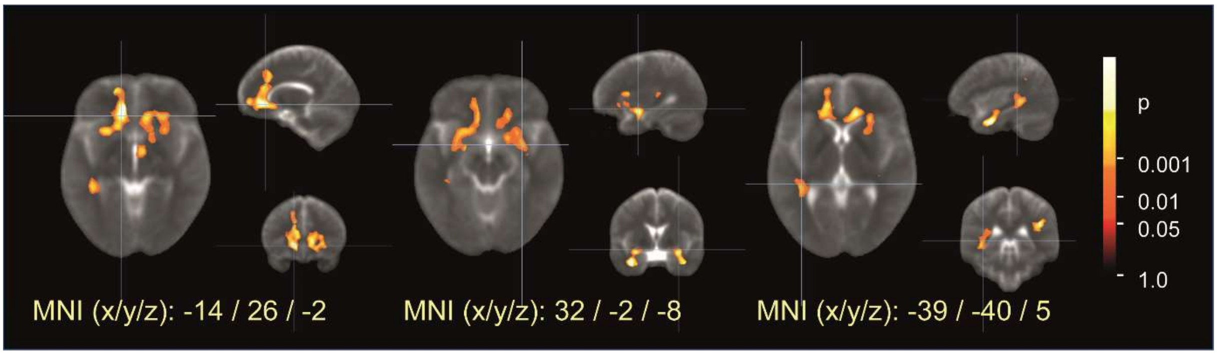

Supplementary Figure 1: Whole brain-based spatial statistics (WBSS) of FA maps at the group level for 9 adolescent and adult PMS patients (14-56 years old) vs 9 matched controls. WBSS of FA maps demonstrated multiple clusters of regional FA reductions at $p<0.05$ (corrected for multiple comparisons, FDR).

Abbreviations: FA = fractional anisotropy; FDR = false-discovery-rate; MNI = Montreal Neurological Institute; PMS = Phelan-McDermid syndrome; WBSS = whole brain-based spatial statistics.
